# Comparison of Good Clinical Practice Inspection Processes for Marketing Applications Between the United States Food and Drug Administration and the European Medicines Agency

**DOI:** 10.1007/s43441-022-00441-w

**Published:** 2022-08-16

**Authors:** Kassa Ayalew, Yang-Min Ning, Michelle J. Foringer, Susan Leibenhaut, Jenn W. Sellers, Bei Yu, Phillip D. Kronstein, Agata Higgerson, Camelia Mihaescu, Miguel Rodriguez, LaKisha Williams, Ni A. Khin

**Affiliations:** 1 Division of Clinical Compliance Evaluation, Office of Scientific Investigations, Office of Compliance, Center for Drug Evaluation and Research, United States (US) Food and Drug Administration (FDA), Silver Spring, MD USA; 2 Inspections Office, Quality and Safety of Medicines Department, European Medicines Agency (EMA), Domenico Scarlattilaan 6, 1083 HS Amsterdam, The Netherlands; 3 U.S. Food and Drug Administration, 10903 New Hampshire Ave, White Oak Building 51, Room 5370, Silver Spring, MD 20993 USA

**Keywords:** Good clinical practice inspection, Inspection process, Clinical investigator, Sponsor, FDA, EMA

## Abstract

**Background:**

The U.S. Food and Drug Administration (FDA) and European Medicines Agency (EMA) began collaboration on Good Clinical Practice (GCP) inspections for marketing applications since 2009. The main characteristics of the GCP inspection processes between FDA and EMA were never evaluated. This is the first analysis comparing the GCP inspection processes between the two agencies.

**Methods:**

We examined and analyzed the key characteristics of the GCP inspection processes, including the geographical distribution, inspection types and timelines from application submission to final inspection reporting for marketing applications from September 2009 through December 2015.

**Results:**

Fifty-five shared applications were included for analysis. For these applications, a total of 433 GCP inspections were conducted in 47 countries. Most clinical investigator (CI) inspections were conducted in regions outside of each agency’s own regulatory jurisdiction, while most sponsor/contract research organization (CRO) inspections were conducted in the U.S. by both agencies. Twenty-eight shared applications included common sites inspected by both agencies. There were 15 joint inspections conducted for seven of these applications and the remaining applications had common sites inspected by both agencies at separate times. Of the joint inspections, 73% were conducted in the U.S and 20% in the E.U. The median time from submission of an application to generation of final inspection reports was 232 days for FDA and 204 days for EMA, with no significant differences noted among applications with and without common sites.

**Conclusion:**

The inspection processes and timelines between the two agencies were similar, providing support for continued FDA-EMA GCP collaboration.

## Introduction

GCP inspections of clinical investigators (CI), sponsors and contract research organizations (CRO) in clinical trials are critical for regulatory assessments of new drug and biologics license applications at both the U.S. Food and Drug Administration (FDA) and European Medicines Agency (EMA). Prior to 2009, both agencies conducted separate GCP inspections for the same marketing applications (term used by FDA) or marketing authorization applications (term used by EMA) during regulatory assessments.

In 2009, FDA and EMA began collaborating on GCP inspection activities under a confidentiality agreement [1]. The collaboration was intended to better understand the GCP inspection operations and regulatory requirements of each agency and to allow for exchange of inspection findings given the increasing number of globalized clinical trials [2]. The collaboration includes bimonthly meetings to discuss the status and/or findings from inspections for shared applications, exchange inspection documents, and identify sites for potential joint or observational inspections. In joint inspections, representatives from both agencies are at the same site and conduct the inspection simultaneously, with each regulatory agency writing a separate inspection report. During a joint inspection, the two agencies share information and documents and collaborate on various aspects of the inspection. During observational inspection, both agencies are at the same site and one regulatory agency conducts the inspection while the other regulatory agency observes similarities and differences in inspection procedures. Each agency may also inspect a common site at separate time, termed sequential inspection, (i.e., each agency conducts a separate official inspection and writes a separate report) due to different timelines. The approach to collaboration and the ability to coordinate inspection planning depended on the timing of the submission to each agency. Differences concerning whether to inspect and what site (CI, sponsor, or CRO) to inspect, the locations of the inspections, and the various regulatory timelines of each agency also contributed to the ability of the two agencies to effectively collaborate in this initiative.

For this report, we examined inspection processes between the two agencies in GCP inspections and evaluated various performance characteristics that potentially impact coordinating inspection planning and exchange of information in this initiative.

## Methods

### Data Sources

Both FDA and EMA use several systems and databases to maintain inspection data related to marketing applications. FDA used the Document Archiving, Reporting, & Regulatory Tracking System (DARRTS), Compliance Program Information System (COMPLIS), and Enterprise Content Management System (ECMS—document repository). The EMA used the product information and application tracking system (SIAMED), and the inspections database (CorpGxP). FDA document the results and findings of a GCP inspection in an Establishment Inspection Report (EIR) and Clinical Inspection Summary while EMA document in an Inspection Report (IR) and Integrated Inspection Report (IIR).

### Identification of Inspection Data and Analyses of Inspection Processes

Inspection data were obtained from the data sources maintained by each respective agency. We first identified the shared applications, defined as an application that the same clinical trial data were submitted to both FDA and EMA for marketing authorization by the same applicant. Shared applications from September 2009 through December 2015 were identified by reviewing meeting agendas and lists of documents that had been exchanged over the course of the FDA-EMA collaboration during the study period. For each of the shared applications identified, the number and type of sites inspected, location of inspected sites, and relevant timelines of inspection process milestones at each agency were examined and tabulated accordingly. Shared applications were further classified into those in which there were common sites inspected by FDA and EMA and those that did not share common sites. For those applications that had common sites, inspections were categorized as joint inspections that were conducted concurrently by both agencies, and sequential inspections that were conducted separately at different times by both agencies. For applications that had no common sites between the two agencies, the sites were designated “FDA Only” or “EMA Only”, as appropriate.

A mapping structure to determine the most logical method of comparing data between the two agencies was developed (Table 1). A data dictionary was developed to ensure that the identified timepoints were correctly labeled and cross-checked for the analysis. An inspection reference number was used to map each EMA inspection to its corresponding FDA inspection in FDA’s COMPLIS database. Once the attributes for analysis were identified, each agency extracted the respective data for each application and conducted several rounds of quality control using the applicable source documentation, including FDA’s EIR and Inspection Assignment Memo and EMA’s IIR and inspection announcement letter. EMA’s data was then imported into FDA’s COMPLIS database, and a report was generated to combine each agency’s corresponding inspection data for analysis.

**Table 1:** Data Sources for Analysis of GCP Inspection characteristics and Timelines

Characteristics of Timeline	FDA Data Source	EMA Date Source
Submission date	Receipt date for submission in DARRTS	Date of validated submission based on SIAMED
Consult date for inspection	Consult and requested inspection sites signed in DARRTS	First adoption of inspection request by CHMP based on CorpGxP
Assignment issuance date for inspection	Assignment documented in COMPLIS	Announcement letter in CorpGxP
Inspection start and end date	Establishment inspection report	Integrated inspection report based on CorpGxP
Inspection report date	Clinical inspection summary	Integrated inspection report based on CorpGxP
Regulatory action date	Date of action letter in DARRTS	Date of CHMP opinion or withdrawal letter

*DARRTS* Document Archiving, Reporting and Regulatory Tracking System, *COMPLIS* compliance program information system, *SIAMED* Sistema de Información Automatizada sobre Medicamentos, *CHMP* Committee for Medicinal Products for Human Use, *CorpGxP* corporate GxP database

### Study Outcomes

We used descriptive statistics to tabulate the identified inspection characteristics and analyze timelines related to these characteristics (Table 2) between FDA and EMA. For applications that had two or more inspection assignments during the review course, relevant timelines were calculated based on the date of the first assignment, first inspection start, last inspection, and final inspection report regardless of the assignment sequence.

**Table 2:** Distribution of Clinical Investigator and Sponsor/Contract Research Organization (CRO) Inspections by Agency and Respective Regulatory Region

	Agency	In the U.S	In the E.U.^a^	Others^b^
Clinical investigator (*n* = 345)	FDA	103 (47%)	55 (25%)	61 (28%)
	EMA	30 (24%)	31 (25%)	65 (51%)
Sponsor/CRO (*n* = 88)	FDA	42 (82%)	6 (12%)	3 (6%)
	EMA	22 (59%)	12 (33%)	3 (8%)

^a^Inspections conducted in 18 countries of the European Union (2009–2015): Austria, Belgium, Bulgaria, Croatia, Czechia, Estonia, France, Germany, Greece, Hungary, Italy, Latvia, Netherlands, Poland, Romania, Spain, Sweden, and United Kingdom

^b^Others include inspections in 27 countries and one region outside the U.S and E.U.: Argentina, Australia, Brazil, Canada, Chile, China, Colombia, Dominican Republic, Ecuador, Egypt, Georgia, India, Israel, Japan, Malaysia, Mexico, New Zealand, Norway, Peru, Republic of Korea, Republic of Serbia, Russian Federation, South Africa, Switzerland, Taiwan, Thailand, Turkey, and Ukraine

## Results

### General GCP Inspection Characteristics of the Shared Applications

**Fig. 1:**
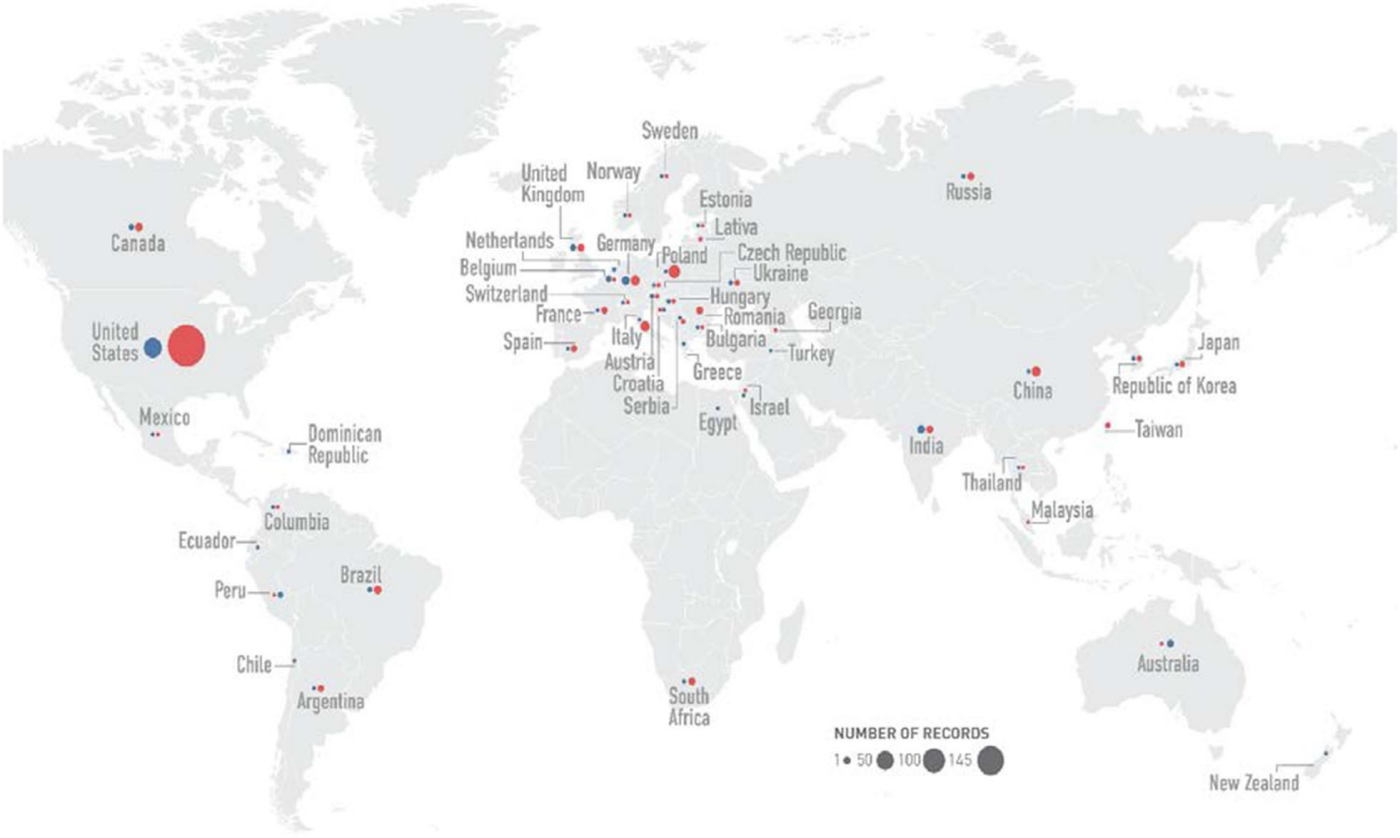
Global Distribution of Clinical Inspections Conducted by FDA and EMA for Shared Applications. Red dots denote inspections by the US FDA; Blue dots denote inspections by EMA

Fifty-nine shared applications had GCP inspections by both FDA and EMA from September 2009 through December 2015. Four applications were excluded from the analysis. One application was refused to file by FDA after submission. Three other applications had trial data from different study protocols. For the 55 shared applications included in the analysis, a total of 433 GCP inspections were conducted in 47 countries across the globe (Fig. 1), with 45% in the U.S., 25% in European Union (E.U.), and 30% in other countries and regions outside the U.S and E.U. Of these inspections, 345 (80%) were for CIs and 88 (20%) for sponsors/CROs (Table 2). For the CI inspections, almost half conducted by FDA were located in the U.S. (103 out of 219, 47%) while about three quarters conducted by EMA were located outside of E.U. (95 out of 126, 75%). The median number of CI inspections per application was four for FDA and two for EMA, respectively. For the sponsor/CRO inspections, the majority of the inspections were conducted in the U. S. (Table 2).

Of all the inspections conducted, 31% were for marketing applications in oncology and hematology, 20% in cardiology, neurology, and nephology, 14% for in infectious diseases, 11% in endocrinology, and 24% in other disease areas (e.g., rheumatology, gastroenterology, urology, psychiatry, dermatology, and medical imaging).

### Characteristics of GCP Inspections Conducted for Shared Applications with Common Sites

Twenty-eight out of 55 (51%) shared applications had 43 common sites inspected by both agencies. These included 23 CI sites and 20 sponsors/CROs (Table 3). This represents 20% of the total 433 inspections conducted by both agencies. Of the 43 common sites, 15 sites (ten CIs and five sponsors) had joint inspections conducted by FDA and EMA concurrently for seven applications; 28 sites (13 CIs and 15 sponsors/CROs) were inspected sequentially by both agencies for 21 applications. Most joint inspections were conducted in the U.S., with 70% (14 out of 20) for CIs and 80% (8 out of 10) for sponsors/CROs. Thirty one percent (8 out of 26, 31%) of sequential inspections for common CI sites were conducted in the US while most of common sponsor/CRO sequential inspections (22 out of 30, 73%) occurred in the U.S. (Table 3).

**Table 3:** Distribution of Inspections for Shared Applications with and without Common Sites by Agency and Respective Regulatory Region

Distribution of Inspections	Clinical Investigators			Sponsors/Contract Research Organizations		
	In the U.S	In the E.U	Others	In the U.S	In the E.U	Others
Joint inspections (common sites) *n* = 30	14 (70%)	4 (20%)	2 (10%)	8 (80%)	2 (20%)	0
Sequential inspections (common sites) *n* = 56	8 (31%)	4 (15%)	14 (54%)	22 (73%)	6 (20%)	2 (7%)
Inspections by FDA only *n* = 227	92 (47%)	51 (26%)	53 (27%)	26 (84%)	3 (10%)	2 (6%)
Inspections by EMA only *n* = 120	19 (19%)	27 (26%)	57 (55%)	8 (47%)	7 (41%)	2 (12%)

### Characteristics of GCP Inspections Conducted for Shared Applications Without Common Sites

Twenty-seven out of 55 shared applications (49%) had no common sites inspected by both agencies. Of the total 347 inspections for these applications, 227 were conducted by FDA only and 120 by EMA only (Table 3). FDA conducted 104 (53%) CI inspections outside the U.S. versus 92 (47%) in the U.S., while EMA had 76 (74%) outside the E.U. and 27 (26%) in the E.U. US conducted the majority of sponsor/CRO inspections (84%) in the U.S. while EMA conducted about half (47%) in the U.S. (Table 3).

### GCP Inspection Processes and Related Timelines

For all the shared applications, both agencies completed GCP inspections prior to taking regulatory actions. Overall, the time interval from the submission of the applications to generation of final inspection reports was similar between the two agencies, with a median time of 232 days for FDA and 204 days for EMA (Table 4). Compared between the applications with and without common sites, median times were also similar: 241 days with common sites versus 232 days without common sites for FDA; 208 days with common sites versus 197 days without common sites for EMA (Table 4).

**Table 4:** Timelines of Inspection Processes for Shared Applications between FDA and EMA

	Timeline Median Day (Range)					
	Overall		With Common Sites		Without Common Sites	
	FDA	EMA	FDA	EMA	FDA	EMA
Application submission to final inspection report	232 (134, 448)	204 (111, 557)	241 (144, 448)	208 (167, 486)	232 (134, 314)	197 (111, 557)
Selection of site after submission	59 (− 15, 224)	36 (19, 448)	61 (17, 224)	49 (22, 338)	53 (− 15, 126)	36 (19, 448)
Assignment to field inspection	15 (− 1, 176)	12 (− 27, 43)	15 (1, 176)	13 (− 27, 43)	11 (− 1, 161)	11 (1, 29)
Initiation of inspection after assignment	34 (3, 134)	59 (18, 201)	32 (7, 106)	55 (18, 109)	40 (3, 134)	60 (32, 201)
Completion of inspection	47 (1, 128)	32 (3, 157)	44 (1, 128)	32 (4, 157)	46 (1, 122)	31 (3, 74)
Inspection reporting after last inspection	46 (− 4, 167)	47 (8, 161)	47 (6, 167)	47 (8, 161)	43 (− 4, 137)	48 (19, 108)

Five key inspection process timelines were evaluated, and the results are listed in Table 4. Overall, median times spent for Selection of Inspection Entities after Submission (59 days for FDA and 36 days for EMA) and Completion of Inspections (47 days for FDA and 32 days for EMA) were slightly shorter for EMA, whereas median time for Initiation of First Inspection after Assignment (34 days for FDA and 59 days for EMA) was slightly shorter for FDA. Median times for Assignment to Field and Inspection Reporting after Last Inspection were highly similar between two agencies. For each agency, there were no notable differences in these sub-timelines between the shared applications with and without common sites.

## Discussion

This is the first analysis of the GCP inspection process comparing timelines between FDA and EMA since the two agencies established GCP inspection collaboration in 2009. The results show that for the 55 shared applications, each agency conducted hundreds of GCP inspections across the world. Most of the clinical investigator inspections were conducted in regions outside of each agency’s respective regulatory territory, whereas most sponsor/CRO inspections occurred in the U.S. This probably was due to the fact that the shared applications were mostly global trials and the majority of sponsors/CROs were based in U.S. Twenty percent of the total inspections were performed by both agencies at the common inspection sites, either jointly or sequentially. In general, final inspection reports were completed approximately 7 months following submission of an application for both agencies. This result reveals that the overall GCP inspection process timelines (e.g., length of time from application submission to final inspection reporting) are not considerably different between the two agencies. For example, from the shared applications included in the pilot, the FDA took two to 3 weeks longer than the EMA to select inspection sites and complete inspections even though FDA initiated the inspections about 3 weeks sooner after assignments. This could be due to factors such as FDA generally conducted more CI inspections than EMA (four CI inspections for FDA versus two for EMA), and therefore, it took longer for FDA to complete all inspections.

The key limitation of this study is the small number of the shared applications identified during the study period. We examined only inspections conducted by the two agencies during the study period. This may not well reflect increasing GCP collaborative activities of the two agencies in more recent years. Another limitation is the retrospective nature of data collection and the fact that the analysis of these shared applications occurred in many cases, several years after application submission. This retrospective approach made it difficult to fully understand differences in the method by which each agency selected sites to inspect.

Overall, our study shed light on differences and similarities in GCP inspection process timeline between the two agencies for the inspections conducted for the shared applications between 2009 and 2015. The observed inspection timelines suggest that both agencies have similar GCP inspection processes. The minimal differences in timelines did not appear to affect the overall completion of GCP assessments by each agency.

Our recent study outlined the similarities and differences of GCP inspection findings from both agencies. The results showed that GCP inspection findings from common CI and sponsor/CRO inspections were comparable with respect to protocol compliance and trial management, providing support for continued FDA-EMA GCP collaboration [3].

FDA and EMA have existing programs in place to evaluate compliance with applicable regulatory requirements and GCP provisions [4–10]. Until 2009, these programs had neither included a bilateral, systematic coordination in conduct of GCP inspections for marketing applications of common interest nor developed a systematic and timely mechanism for sharing relevant GCP-related information.

## Conclusion

The inspection processes and timelines between the two agencies were similar, providing support for continued FDA-EMA GCP collaboration. The similar timelines allow for more efficient use of finite resources, reduce duplicative inspections, and broaden inspection coverage by the two regulatory agencies. This is especially important when a shared application is submitted to both agencies around the same time, permitting for real time information sharing and collaborative inspections.
